# Health practitioners' perceptions of the barriers and enablers to the implementation of reproductive genetic carrier screening: A systematic review

**DOI:** 10.1002/pd.5914

**Published:** 2021-03-05

**Authors:** Stephanie Best, Janet Long, Tahlia Theodorou, Sarah Hatem, Rebecca Lake, Alison Archibald, Lucinda Freeman, Jeffrey Braithwaite

**Affiliations:** ^1^ Australian Institute of Health Innovation Macquarie University Sydney New South Wales Australia; ^2^ Australian Genomics Health Alliance Murdoch Children's Research Institute Melbourne Victoria Australia; ^3^ Victorian Clinical Genetics Services Murdoch Children's Research Institute Royal Children's Hospital Melbourne Victoria Australia; ^4^ Bruce Lefroy Centre Murdoch Children's Research Institute Royal Children's Hospital Melbourne Victoria Australia; ^5^ Department of Paediatrics Faculty of Medicine, Dentistry and Health Sciences University of Melbourne Parkville Victoria Australia; ^6^ Centre for Clinical Genetics Sydney Children's Hospital Randwick Randwick New South Wales Australia; ^7^ School of Women's and Children's Health University of New South Wales Randwick New South Wales Australia

## Abstract

**Background:**

As interest in reproductive genetic carrier screening rises, with increased availability, the role of healthcare practitioners is central in guiding uptake aligned with a couples' values and beliefs. Therefore, practitioners' views on implementation are critical to the success of any reproductive genetic carrier screening programme.

**Aim:**

To explore healthcare practitioners' perceptions of the barriers and enablers to implementation.

**Materials & Methods:**

We undertook a systematic review of the literature searching seven databases using health practitioner, screening and implementation terms returning 490 articles.

**Results:**

Screening led to the inclusion of 26 articles for full‐text review. We found three interconnected themes relating to reproductive genetic carrier screening: (i) use and impact, (ii) practitioners' beliefs and expectations and (iii) resources.

**Discussion:**

Barriers and enablers to implementation were present within each theme and grouping these determinants by (a) community for example lack of public interest, (b) practitioner for example lack of practitioner time and (c) organisation for example lack of effective metrics, reveals a preponderance of practitioner barriers and organisational enablers. Linking barriers with potential enablers leaves several barriers unresolved (e.g., costs for couples) implying additional interventions may be required.

**Conclusion:**

Future research should draw on the findings from this study to develop and test strategies to facilitate appropriate offering of reproductive genetic carrier screening by healthcare practitioners.

## Key Points

**What is already known?**

- Availability of reproductive genetic carrier screening is rising.
- Screening is often focused on ethnically specific conditions or for those with a family history of disease.
- Commonly, carriers do not have family history of disease.

**What does this review add?**

- Identifies practitioner barriers to implementation of reproductive genetic carrier screening, for example, beliefs and expectations.
- Matches identified practitioner barriers to enablers to implementation.
- Highlights where additional implementation support is required, for example, lack of practitioner confidence.

## 1 INTRODUCTION

Internationally, the move towards population level reproductive genetic carrier screening (RGCS) of prospective parents to identify their risk of having a child with a genetic condition, is growing. Technological advances are making the process more feasible and costs are falling.^1^ Despite the routine practice of RGCS for ethnically specific conditions, such as thalassaemia or Tay–Sachs disease, or in circumstances where there is a family history of disease, healthcare practitioners' (HCPs) attitudes towards screening in the general ‘low‐risk’ population remain unclear. It is well documented^2^ that there is no family history in approximately 88% of carriers, emphasising the importance of offering RGCS to all couples planning for a child and guidelines are evolving to reflect this.

To date, attention in the literature has centred on the attitudes of patients and families of those affected by genetic conditions,^3^, ^4^, ^5^ hypothetical views of HCPs on whether screening should be offered^6^ and the cost effectiveness of RGCS.^7^, ^8^ While these elements are important, they do not inform us about how to implement a RGCS programme. The study of implementation facilitates the focus on the factors associated with the success or failure of a clinical intervention.^9^

Fortunately, there is now an emerging evidence base identifying HCPs perceptions of factors influencing the implementation of RGCS. To the best of our knowledge, a systematic review of the literature in this area has not been undertaken. As many countries are investing in the use of RGCS,^10^ it is timely to investigate factors influencing its implementation. As patients should be made aware of RGCS, so that they can use RGCS according to their values and beliefs,^11^ this phase is an essential first step to identifying appropriate strategies to support HCPs offering RGCS in their day‐to‐day practice.

## 2 AIMS AND OBJECTIVES

The aim of this systematic review was to identify barriers and enabling factors associated with the implementation of RGCS particularly in relation to the views of HCPs. The study had the following objectives:

- To examine HCPs perceptions of barriers and enablers to the implementation of reproductive genetic carrier screening.
- To investigate HCPs attitudes towards the implementation of reproductive genetic carrier screening.
- To reveal areas where further primary research is required.

## 3 METHODS

The literature search was registered with PROSPERO (registration number CRD42020150581) and conducted in September 2020. Preferred Reporting Items for Systematic Reviews and Meta‐Analyses guidelines were followed^12^ and the databases Medline, EMBASE, Scopus, PsycINFO, Web of Science, PubMed, and CINAHL were searched. Search terms were chosen through exploration of MeSH terms, consideration of key words in current RGCS articles and suggestions of expert researchers in the field. Search terms included ([‘health personnel’ OR [‘attitude of health personnel’ OR ‘healthcare providers’ OR ‘general practitioners’ OR ‘obstetricians’ OR ‘gynaecologists’] AND [‘mass screening’ OR ‘carrier screening’ OR ‘genetic screening’ OR ‘genetic testing’] AND [‘reproductive’ OR ‘prenatal’ OR ‘preconception’ OR ‘antenatal’ OR ‘universal’ OR ‘expanded’ OR ‘autosomal recessive’ OR ‘x‐linked’ OR ‘cystic fibrosis’ OR ‘fragile x’ OR ‘spinal muscular atrophy’ OR Duchennes muscular dystrophy OR thalassaemia] AND [‘implementation science’ OR ‘implementation’]). Articles were downloaded into Endnote X9, a bibliographic database. Duplicates and incomplete references were discarded resulting in 490 papers for inclusion. We also used a snowball process for cited articles from the initial search to generate another 28 papers.

Five reviewers (J.C.L., T.T., S.B., R.L. and S.H.) analysed the same 10% of titles and abstracts independently, applying inclusion and exclusion criteria (Table 1). We included empirical, human research and excluded any guidelines, commentaries, opinion pieces and studies using secondary data. Only studies related to HCPs engaged with RGCS during the prepregnancy or prenatal period were included. General public and target population views were not included.

**TABLE 1:** Inclusion and exclusion criteria for articles

Inclusion criteria	Exclusion criteria
Peer‐reviewed empirical research	Opinion pieces, comments, editorials, etc. (not empirical research)
Reproductive genetic carrier screening	Other prenatal screening, e.g., NIPT/chromosomal
Humans	Animal models
Implementation or preimplementation of screening programs in a ‘real world’ context	Nonclinical research without a link to a clinical ‘real‐world’ context (e.g., studies investigating which conditions to be included in reproductive carrier screening panels)
Focus is on health care practitioners' attitudes, in the context of identifying barriers and enablers to implementation	Focus not on health care practitioners' attitudes
Health practitioners engaged with reproductive genetic carrier screening	General public and client views

Abbreviation: NIPT, noninvasive prenatal test.

**FIGURE 1:**
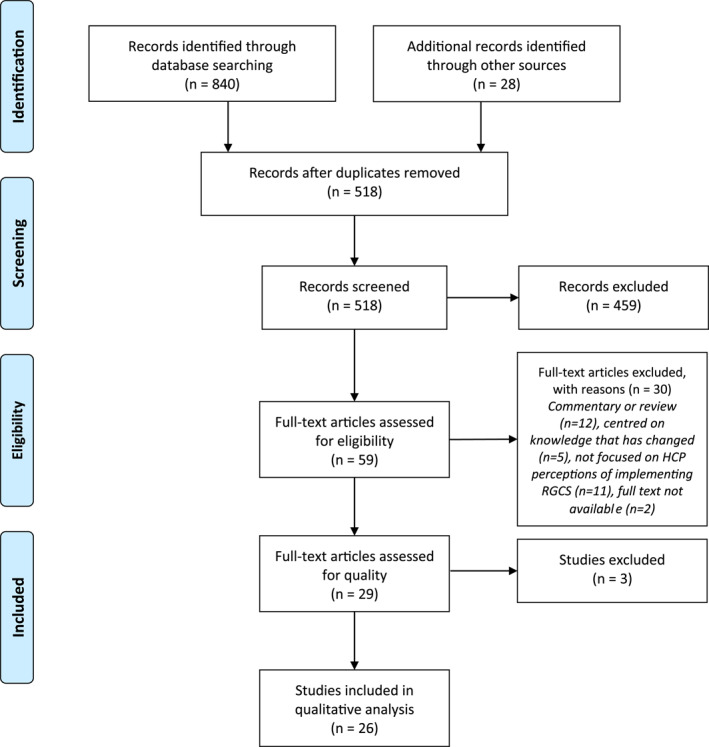
PRISMA flow diagram. PRISMA, Preferred Reporting Items for Systematic Reviews and Meta‐Analyses

Results of the title and abstract screening were compared, and the Fleiss' Kappa statistic was determined to measure inter‐rater reliability, achieving *k* = 0.79 which is interpreted as ‘substantial agreement’.^13^ Following this assessment, the remaining 440 articles were screened independently with the five authors (J.C.L., T.T., S.B., R.L. and S.H.) screening 55 articles each with discussion across the team regarding any challenging articles. The full‐text of the resulting 59 articles were assessed (S.B. and J.C.L.), with 30 discarded as not meeting the inclusion criteria. The remaining 30 were further assessed for quality using the Hawker Tool^14^ with three articles discarded on the basis of poor reporting of bias and ethical issues. The final 26 full‐text articles to be included in the review were then analysed. The full search strategy is shown in Figure 1.

### 3.1 Data analysis

We imported the final 26 papers into NVivo 12^15^ to facilitate data management. Using Braun and Clarke's^16^ approach to thematic analysis, thereby bringing structure to the analysis, two authors (S.B. and J.C.L.) familiarised themselves with the papers before independently assessing eight papers to identify codes in the data. Through a series of discussions, we developed three themes and one author (S.B.) then completed the coding with ongoing discussion with the team. We further compiled the findings from the themes by barriers and enablers for the community, practitioners and organisations.

## 4 RESULTS

First, we outline the types of papers found from the literature search (Table 2) before sharing the themes and subthemes identified (Table 3). Barriers and enablers identified for each subtheme are presented.

From the 26 papers in our search, eight were published before 2010, eight between 2010 and 2015 and 10 from 2016 onwards. The United States of America (n = 8), the Netherlands (n = 7) and Australia (n = 7) returned the most articles with two from the United Kingdom, one from Belgium and one from Sweden. The majority of participants (n = 16) were nongenetic clinicians (primary care e.g., general practitioners [GPs] n = 6; secondary care e.g., obstetricians, n = 7 and; both n = 3) with 10 papers including genetic professionals. Sixteen of the papers were set in the context of RGCS for a specific condition for example fragile X, nine were based on expanded RGCS and one considered both. Quantitative studies made up the mainstay of the methods (n = 14), with 11 qualitative studies and one mixed method.

**TABLE 2:** Characteristics of final full text articles included

References	Country	Condition(s) screened	Aim	Methodology	Participants
Archibald et al.^21^	Australia	Fragile X	To explore the attitudes of health practitioners, relatives of people with fragile X syndrome, potential target populations for carrier screening (pregnant and nonpregnant women), and the general community regarding population‐based carrier screening for fragile X syndrome.	Interviews and focus groups	Both primary and secondary health care providers
Archibald et al.^29^	Australia	Fragile X	To explore stakeholder views about offering population‐based genetic carrier screening for fragile X syndrome.	Interviews and focus groups	Both primary and secondary health care providers, including genetic professionals
Baars et al.^36^	The Netherlands	Cystic fibrosis (CF)	To investigate genetic knowledge and genetic testing among general practitioners (GPs), gynaecologists (GYNs) and paediatricians in the Netherlands.	Surveys	Both primary and secondary health care providers, including genetic professionals
Benn et al.^37^	USA	Expanded	To assess the opinions of fellows of the American College of obstetricians and GYNs on expanded carrier testing and noninvasive prenatal testing.	Surveys	Secondary
Briggs et al.^48^	USA	Expanded	To assess current practice utilisation and attitudes regarding the implementation of expanded carrier screening (ECS) into clinical practice.	Surveys	Secondary
Cho et al.^30^	USA	Expanded	To clarify genetic practitioners' views on the potential benefits and challenges of ECS. To solicit advice from genetics practitioners regarding how best to integrate ECS into preconception reproductive healthcare.	Focus groups	Secondary—genetic professionals
Cousens et al.^31^	Australia	β‐Thalassaemia	To gain a better understanding of healthcare practitioners' practice and attitudes regarding prenatal β‐thalassaemia carrier screening in Australia.	Interviews	Both primary and secondary health care providers, including genetic professionals
Cunningham et al.^17^	Australia	CF	To investigate the attitudes of healthcare practitioners caring for patients with CF toward population‐based carrier screening for CF.	Surveys	Primary
Darcy et al.^18^	USA	CF	To determine the current awareness by obstetricians of the existence and content of practice guidelines, the variety in practice regarding CF carrier screening, and the level of knowledge regarding CF genetics and screening result interpretation. To explore potential barriers to offering screening and whether academic affiliation or type of practice influences outcome.	Surveys	Secondary
Delgado et al.^40^	USA	Preconception genetic screening	To assess provider knowledge, comfort with counselling, formal training and educational needs regarding prenatal genetic screening and diagnostic testing.	Surveys	Secondary, including genetic professionals
Holtkamp et al.^27^	The Netherlands	Expanded	To identify general and population‐specific barriers and needs reflected by Dutch stakeholders regarding the implementation of (expanded universal) carrier screening.	Interviews	Both primary and secondary health care providers, including genetic professionals
Janssens et al.^32^	Belgium	Expanded	To explore attitudes of clinical and molecular geneticists about the implementation of multidisease or ECS for monogenic recessive disorders.	Interviews	Secondary—genetic professionals
Janssens et al.^19^	Belgium	Expanded	To explore European geneticists' attitudes towards ECS, focussing on their concrete suggestions and recommendations for the use of ECS in the clinical setting.	Interviews	Secondary—genetic professionals
Lazarin et al.^34^	USA	Expanded	To conduct an extensive survey in a large genetic counsellor population on personal and practitioner attitudes regarding ECS.	Surveys	Secondary—genetic professionals
Matar et al.^23^	Sweden	Expanded	To explore and describe Swedish healthcare practitioners' perceptions of preconception ECS with focus on the ethical aspects.	Interviews	Secondary, including genetic professionals
McClaren et al.^24^	Australia	CF	To explore whether or not CF carrier screening should be offered in the Australian setting, the best time for offering carrier screening, the information required for making a decision about carrier screening, and how this information can best be provided.	Interviews and focus groups	Both primary and secondary health care providers
Morgan et al.^33^	USA	CF	To increase understanding of when, how, and in what populations obstetrician‐GYNs are implementing the published guidelines for CF carrier screening and to learn the physicians' opinions and practices regarding CF screening.	Surveys	Secondary
Morgan et al.^39^	USA	CF	To assess the impact of self‐reported familiarity with published guidelines on knowledge, implementation, and opinions of obstetrician–GYNs regarding carrier screening for CF.	Surveys	Secondary
Poppelaars et al.^28^	The Netherlands	CF	(1) To investigate the attitude of potential providers (GPs and MHS workers) and the target population (couples planning a pregnancy) towards a CF carrier screening programme; (2) to investigate opinions regarding the preferred method of implementation; (3) to determine the role of GPs and MHS workers in the screening and pre‐test education, and (4) to determine the support needed.	Surveys	Both primary and secondary health care providers
Poppelaars et al.^25^	The Netherlands	CF	To explore the possibilities and barriers in the implementation of a nationwide preconceptional CF carrier screening programme in The Netherlands.	Focus groups	Both primary and secondary health care providers
Poppelaars et al.^35^	The Netherlands	CF	To investigate the attitudes of GPs and CHS workers with regard to routinely offering preconceptional CF carrier screening, and to identify variables which were associated with a positive and a negative attitude.	Surveys	Both primary and secondary health care providers
Qureshi et al.^38^	UK	CF and thalassaemia	To assess GPs' confidence in their ability to provide initial prenatal advice for couples carrying common autosomal recessive disorders (either the CF or thalassaemia gene), and their opinions of different approaches for referral to prenatal diagnostic services for such at‐risk couples.	Surveys	Primary
Schuurmans et al.^7^	The Netherlands	Expanded	To investigate whether test‐provision by GPs could be a feasible approach for ECS and result in informed choice of couples who attended pretest counselling.	Checklist and interviews	Primary
Stark et al.^26^	Australia	General including β‐ thalassaemia, CF and fragile X	To gather information about the current practice and attitudes of Australian obstetricians toward carrier screening for genetic conditions as part of routine pregnancy care.	Surveys	Secondary
Tsianakas et al.^22^	UK	Sickle cell disease and thalassaemia	To assess the feasibility of offering antenatal SC&T screening in primary care at the time of pregnancy confirmation.	Interviews	Primary
Valente et al.^20^	Australia	CF	To explore the opinions, knowledge and practice patterns of GPs, obstetricians and fertility specialists in Victoria, Australia.	Survey	Both primary and secondary health care providers

Abbreviations: CHS, Community health service; MHS, Municipal health services; SC&T, sickle cell and thalassaemia.

**TABLE 3:** Themes and subthemes identified with papers referring to themes. Key: Papers referring to expanded reproductive carrier screening are prefixed with E, papers with a focus on the subtheme are underlined

Theme	Subtheme	Papers
(i) The use and potential impact of reproductive genetic carrier screening	Achieving equitable service provision, including cost to the individual	Archibald (2016)^29^ ; Cunningham (2014)^17^ ; Darcy (2011)^18^ ; E Holtkamp (2017)^27^; E Janassens (2017)^19^; E Matar (2016)^23^; Tsianakas (2010)^22^; Valente (2020)^20^
	Potential impact (including the offer) on the client, including concern about client anxiety, informed choice and stigma	Archibald (2012)^21^ ; Archibald (2016)^29^ ; Cousens (2014)^31^ ; Cunningham (2014)^17^; E Cho (2013)^30^; E Holtkamp (2017)^27^; E Janssens (2017)^19^; E Janssens (2017)^32^; E Matar (2016)^23^; E Schuurmans (2019)^7^; McClaren (2008)^24^; Poppelaars (2003)^25^; Poppelaars (2003)^28^; Stark (2013)^26^; Tsianakas (2010)^22^
(ii) Practitioner beliefs and expectations about the process of delivering reproductive genetic carrier screening	Practitioner attitudes to and beliefs about RGCS	Archibald (2012)^21^; Archibald (2016)^29^; Baars (2004)^36^; Cunningham (2014)^17^; E Holtkamp (2017)^27^; E Janssens (2017)^19^; E Janssens (2017)^32^; E Lazerin (2016)^34^; E Matar (2016)^23^ ; E Schuurmans (2019)^7^; McClaren (2008)^24^; Morgan (2004)^33^; Poppelaars (2003)^28^; Poppelaars (2004)^35^; Stark (2013)^26^; Tsianakas (2010)^22^; Valente (2020)^20^
	Practitioner perceptions of their ability to deliver RGCS including client selection, interpreting results and confidence	Archibald (2012)^21^; Archibald (2016)^29^; Darcy (2011)^18^; E Benn (2014)^37^; E Cho (2013)^30^; E Janssens (2017)^19^; E Matar (2016)^23^; E Schuurmans (2019)^7^; Morgan (2004)^33^; Morgan (2005)^39^; Qureshi (2005)^38^; Stark (2013)^26^; Tsianakas (2010)^22^
	Practitioner knowledge and support required to deliver RGCS	Cousens (2014)^31^; Cunningham (2014)^17^; Darcy (2011)^18^; Delgado (2020)^40^; E Cho (2013)^30^ ; E Matar (2016)^23^; McClaren (2008)^24^; Morgan (2005)^39^; Valente (2020)^20^
	Practitioner expectations and external views influencing their clinical decision making including the impact of their recommendations, professional bodies, legal expectations	E Benn (2014)^37^; E Cho (2013)^30^; Delgado (2020)^40^; E Janssens (2017)^19^; E Janssens (2017)^19^; E Lazerin (2016)^34^; E Matar (2016)^23^; McClaren (2008)^24^; Morgan (2004)^33^; Poppelaars (2003)^25^; Stark (2013)^26^; Tsianakas (2010)^22^
(iii) Resources available for practitioners for reproductive genetic carrier screening	Provision of counselling including genetic counsellors and other professionals	Darcy (2011)^18^ ; E Benn (2014)^37^; E Cho (2013)^30^; E Janssen (2017)^19^; E Lazerin, (2016)^34^; E Mater (2016)^23^; E Schuurmans (2019)^7^; Poppelaars (2003)^25^; Valente (2020)^20^
	Variation in potential models of service provision including who provides RGCS and when	Archibald (2012)^21^; Baars (2004)^36^; E Holtkamp (2017)^27^; Janssens (2017)^32^; E Matar (2016)^23^; E Schuurmans (2019)^7^; Poppelaars (2003)^25^; Poppelaars (2003)^28^; Stark (2013)^26^; Tsianakas (2010)^22^
	Nonclinical resource barriers including strategic costs, responsibility, time	Archibald (2012)^21^ ; Cousens (2014)^31^; Cunningham (2014)^17^; E Holtkamp (2017)^27^; E Janssens (2017)^19^; E Lazarin (2016)^34^; E Matar (2016)^23^; E Schuurmans (2019)^7^; McClaren (2008)^24^; Poppelaars (2003)^25^ 1; Poppelaars (2003)^28^ 2; Stark (2013)^26^; Tsianakas (2010)^22^; Valente (2020)^20^

*Note*: Papers referring to expanded reproductive carrier screening are prefixed with E, papers with a focus on the subtheme are underlined.

Abbreviation: RGCS, reproductive genetic carrier screening.

### 4.1 Themes

Three themes were identified: (i) the use and potential impact of RGCS, including factors influencing equity of service take up and focus on the client; (ii) practitioners' beliefs and expectations about the process of delivering RGCS, including the ability to deliver RGCS, knowledge about and support for RGCS, opinions about RGCS, and external influences on practitioners; and (iii) resources available for practitioners for RGCS, including counselling, models of care delivery and other nonclinical barriers to delivery of RGCS. Table 3 provides a list of papers referencing each theme.

The most and least frequently discussed subthemes (by number of papers) were found in theme (ii) practitioner beliefs and expectations about delivery of RGCS. Most common was the subtheme ‘Practitioner attitudes to and beliefs about RGCS’, while the least discussed subtheme was ‘Practitioner knowledge and support required to deliver RGCS’.

(i) *Use and potential impact of* *RGCS*. Data for this theme centred on either (a) achieving equitable service provision or (b) potential impact of RGCS (including the offer) on the client.

(a) *Achieving equitable service provision*: Inequitable access to RGCS was a concern and seen as a barrier to implementation in several papers, including the cost of the test to individuals.^17^, ^18^, ^19^, ^20^ Routes to achieving equitable service provision included offering RGCS alongside other health interventions, to promote a wide uptake of testing,^21^ and communication with policy makers and other stakeholders which was seen as essential as the availability of RGCS develops.^22^ There was also discussion about the range of diseases screened for with Matar et al.^23^ noting that private companies were expanding panels beyond the public offer, suggesting this would either promote inequity or stimulate policy development.

(b) *Potential negative impacts of RGCS (including the offer) on the client*: The possibility of adverse impacts from RGCS were noted as a barrier to implementation, with several authors highlighting discussion about raising women's anxiety^19^, ^21^, ^24^, ^25^ just by offering the test,^21^ referring to undue anxiety as ‘collateral damage’^19^ (p. 65). Additionally, the risk of stigma and labelling was identified,^17^, ^23^, ^26^ ‘*it will change the way they see themselves*’^21^ p. 53, though they signpost to additional literature, not sourced in this review, countering this view. Some authors reported the general public has a lack of interest in RGCS^27^, ^28^ or have misguided understandings of creating the ‘perfect child’.^28^ Many papers outlined strategies to overcome these barriers: for example, the importance of informed consent, with clients understanding the implications of RGCS, stressing how active this process needed to be (information giving vs. active decision making)^19^, ^22^, ^29^, ^30^, ^31^ and not feeling coerced.^32^ Tools such as decision aids and client education that facilitate a choice aligned with a couples' values and beliefs were identified as essential to ensure appropriate implementation of RGCS.^22^, ^26^, ^32^ Additionally, the need to improve the knowledge of RGCS of the general population was noted^7^, ^19^, ^25^ with GPs reporting a couple's knowledge of RGCS was reflected in the time required at consultation^7^ as people without knowledge and experience of RGCS can find making a decision as to whether they wish to undergo screening more challenging.^21^

(ii) *Practitioner beliefs and expectations about the process of delivering RGCS*. Four subthemes were identified in this theme: (a) practitioner attitudes to and beliefs about RGCS; (b) practitioner perceptions of their ability to deliver RGCS; (c) practitioner knowledge and support required to deliver RGCS; and (d) practitioner expectations and external views influencing their decision making.

(a) *Practitioner attitudes to and beliefs about RGCS*: Attitudes and beliefs that can act as a barrier to implementation of RGCS included the lack of a collective sense of urgency (i.e., demand from the population and HCPs).^27^ While there was generally a positive attitude towards RGCS^25^ this was not always supported in practice by the offer of screening.^26^ On the other hand, not all practitioners were interested in RGCS, running the risk of inconsistency in practice as to who was offered screening.^7^, ^29^ In addition, there was variability in beliefs about who should be offered testing^19^ with client request reported as the most common reason amongst obstetricians and gynaecologists.^33^ The belief that socioeconomic status would influence the offer of RGCS was raised.^34^ Many HCPs were aware that offering RGCS as routine would influence clients' decisions to take up RGCS which runs counter to informed consent.^22^, ^24^ Some literature discussed the limitations of the gene lists^27^, ^30^ with reported concerns about low test reliability.^35^ Attitudes and beliefs about RGCS included concern about the impact of false positive results,^30^ creating uncertainty especially with results in the ‘grey zone’ that is identifying people carrying ‘*intermediate and small premutation results*’^21^ (p. 53) and about medicalising pregnancy and perceptions of eugenics.^27^ Others discussed unease of including adult onset conditions.^21^, ^23^

Many HCPs hold positive attitudes to RGCS.^20^ GPs recognised they are ideally placed to offer RGCS, as they know their patients and their background, and become more comfortable in raising RGCS once they have experience.^7^

(b) *Practitioner perceptions of their ability to deliver RGCS*: For some HCPs, discussing screening was easier when raised by the patient^36^ though obstetricians and gynaecologists were comfortable with offering RGCS regardless.^33^, ^37^ This ease was not the case for all HCPs, for example, GPs were reported to be concerned about their ability to discuss potential worrying, harmful or high risk results and especially the possibility of pregnancy termination when women are in early pregnancy.^22^ There was also concern about the concept of risk,^21^ lack of confidence in offering prenatal genetic advice,^38^ what diseases to test for, interpreting the results and explaining results^18^, ^23^ and in particular, misunderstanding what a ‘positive’ result means.^18^ Greater confidence in interpreting results and managing positive results was associated with shorter time periods since completion of training.^39^ HCPs perceived their ability to deliver RGCS was hindered by complex and confusing criteria,^33^ a feeling that screening is too hard, and too complicated to answer all patients' questions.^29^ In addition, genetic practitioners' ability to interpret results and provide counselling was limited by beliefs about the quality and size of the gene list.^30^ One enabler identified was training, seen as essential by GPs who, as a result, did not have an issue with managing ‘normal results’ or referring onto genetic services.^7^

(c) *Practitioner knowledge and support required to deliver screening*: While HCPs may be willing to offer screening, they may be limited by their lack of knowledge^20^, ^25^ as a result of their limited training in genetics.^30^ In some cases, HCPs are aware of their limited knowledge and request training.^31^ Enablers to overcome a lack of knowledge may include dissemination of research findings to alleviate HCP concerns about screening and make it more acceptable to them.^24^ It was recognised that there was a need to raise awareness of screening guidelines because familiarity with guidelines was associated with implementation of screening.^18^, ^39^ There was a call for more information, education and support for practitioners around genetics.^18^, ^23^, ^30^

(d) *Practitioner expectations and external views influencing their clinical decision making*: Some HCPs felt driven to offer screening due to concerns about potential liability^33^ and professional obligations^19^ while others were concerned about different laboratories offering different panels and the potential impact this may have on provider liability.^30^, ^37^ Although an enabler to implementation of RGCS, these mechanisms are defensive rather than guided by the needs of the client. However, different legal frameworks in different countries may influence providers' decisions to offer screening or not.^26^, ^32^ There was awareness of multiple stakeholders holding views on the place of RGCS in reproductive care that is constantly being negotiated and renegotiated.^22^ Here, professional bodies were identified as a facilitator, though there is concern when conflicting guidance is offered.^30^, ^37^, ^40^ By way of positive action, there was a call for evidence‐based implementation studying the purpose, potential benefits and risks, relevance and acceptance by society.^23^

(iii) *Resources available for practitioners for RGCS*. Three subthemes were identified within this theme, (a) provision of counselling support including genetic counsellors and other professionals; (b) variation in potential models of service provision; and, (c) nonclinical resource barriers.

(a) *Provision of counselling including genetic counsellors and other professionals*: Several papers identified barriers to implementation centred around support. Counselling is resource intensive,^34^ demanding both skills and time^28^ and many HCPs were aware genetic counsellors are a limited resource.^20^, ^37^ They therefore only make referrals to GCs under specific circumstances.^18^ Although much counselling is undertaken by nonspecialist staff^37^ there was concern that nonspecialist providers would find discussing the implications for pregnancy planning challenging,^19^, ^30^ and potentially underestimate the complexity of genetic counselling.^28^ Enablers noted included ensuring couples were well‐informed before attending a consultation, reducing the time required for genetic counselling,^7^ and noting that over time nonspecialist staff could develop a time efficient routine for successful pretest counselling.^7^

(b) *Variation in potential models of service provision*: GPs were identified as well placed to offer screening although there were barriers identified with keeping up with the knowledge required,^7^ time required and not fitting with GP targets.^22^ However, several papers noted enablers, for example, Schuurmans et al.^7^ mooted the idea of GPs with specialist screening skills, and Tsianakas et al.^22^ considered midwives well placed with more time available. Offering screening outside the medical setting could reduce the medicalisation of pregnancy^26^, ^28^ and the use of repeated visits would ensure testing does not become routine.^19^

The literature also discussed timing of the offer and commonly suggested a preference for offering RGCS prepregnancy to offer clients greater reproductive options,^19^, ^21^, ^25^, ^26^ for example through, as yet unrealised, preconception consultation centres.^19^ Testing interconception was also identified.^27^

(c) *Nonclinical resources*: The literature noted several resource barriers, for example, time for offering and counselling clients^7^, ^17^, ^22^, ^24^, ^25^, ^27^, ^29^, ^34^ and support to overcome language and cultural barriers.^26^ The cost to the health system was identified as a potential challenge^22^, ^23^, ^26^, ^28^ alongside a lack of public health focus on RGCS^27^, ^28^ though acknowledging some countries may have more pressing public health issues or limited resources.^19^ An additional challenge for policy makers was that uptake (a traditional measure of success) cannot be used for RGCS as screening should be voluntary.^19^ The literature identified the need for additional skills training in talking about the test and understanding results.^22^, ^26^, ^27^, ^31^ Furthermore, barriers existed where practitioners lacked incentives to participate and so did not see offering RGCS as part of their role.^28^

To facilitate implementation of RGCS additional resources were noted to potentially alleviate the challenge faced by lack of time.^24^ In addition, the role of leadership was identified as an essential requirement to implementation of a RGCS programme.^27^ Interestingly the need for an implementation plan and appropriate intervention strategies to overcome barriers was highlighted.^25^

## 5 DISCUSSION

**FIGURE 2:**
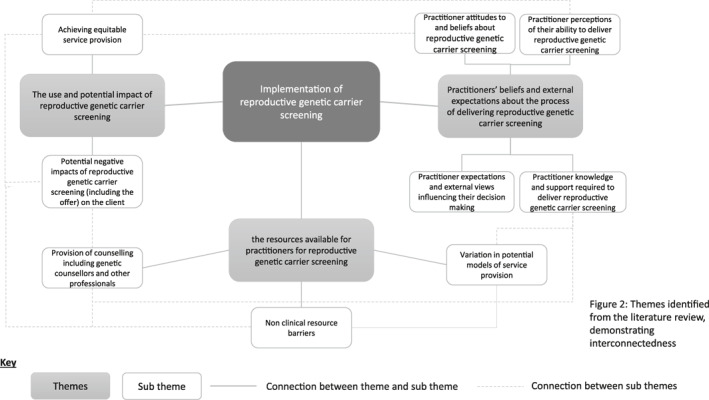
Themes identified from the literature review, demonstrating interconnectedness

The three themes presented in the results (i) the use and potential impact of RGCS; (ii) practitioners' beliefs and expectations about the process of delivering RGCS; and (iii) the resources available for practitioners for RGCS; are highly interconnected via their associated subthemes, as can be seen in Figure 2. For example, practitioner knowledge and support required to deliver RGCS ties across to the provision of counselling, including counsellors and other professions, while achieving equitable service provision is closely linked with the potential impact of RGCS (including the offer) on the patient and practitioner perceptions of their ability to deliver RGCS. These linkages suggest that not only are themes shared but also the barriers and enablers to implementation of RGCS.

Identifying the determinants of implementation is an essential first step in designing implementation strategies to overcome barriers.^41^ Without acknowledging factors that are acting as obstacles, there is a risk of attempting to put in place strategies that do not respond to clinicians' needs and are therefore unlikely to succeed. When examining the themes from the literature it is the barriers that prevail. This may be due to a tendency to focus on negative aspects instead of adopting an approach that encourages a recognition of the learning value of what is going well.^42^ However, there are also enablers in the findings that may inform the development of an implementation strategy.

**FIGURE 3:**
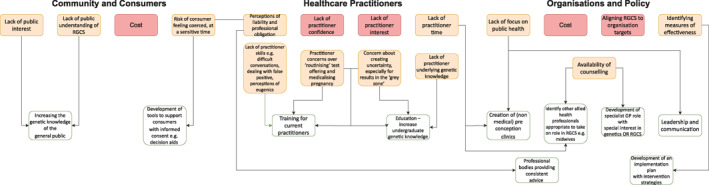
Barriers (yellow) linked with potential enablers (white), identified from the literature – pink boxes indicate no enabler was noted in the literature. Further details can be found in supplementary file

Barriers to the implementation of RGCS identified from the literature are noted in Figure 3 and hypothetically linked with potential enablers. Our search centred on practitioners' perceptions and there is a predominance of practitioner barriers identified. However, interestingly, the majority of the enablers are organisational, suggesting some strategies for implementing RGCS lie beyond the frontline clinicians. It should be stressed that the potential connection of a barrier with an enabler does not mean resolution of the barrier, merely that the literature provides some possible avenues for addressing some of the challenges when implementing RGCS. Some barriers do not have an associated enabler, for example; 1) cost, both to the consumer and the organisation. Addressing this barrier will be highly dependent on the healthcare system in which RGCS is implemented and an essential step in providing an equitable service.^43^ 2) aligning RGCS to organisation targets—this is challenging to address as take‐up rates do not directly equate to the successful provision of a RGCS programme.^19^ and 3) a lack of practitioner confidence and interest—clinician education is clearly an essential first step to overcome these challenges though further interventions may be required for example, peer influence to overcome a lack of interest,^44^ especially as this runs counter to the public interests.^45^ By applying theoretical implementation science and behaviour change approaches, interventions can be designed to overcome these barriers.^46^

## 6 LIMITATIONS

There are limitations with this systematic review. We focused on practitioners' perspectives which, although essential, does not provide the whole picture in regard to implementation, for example, the design of a RGCS programme may influence practitioners' perceptions of the barriers and enablers to implementation. We combined the findings from articles focussing on single gene and expanded RGCS. As the literature develops it may be interesting to identify if some of the barriers and enablers become more significant for expanded RGCS, for example, complexity. The reviewed articles were all based in different healthcare contexts, so we must consider the transferability of the learnings across settings. Additionally, all the studies were based in western countries with no research drawn from lower, and middle‐income countries.

## 7 CONCLUSION

This systematic review identifies HCPs' perceptions of implementation of RGCS programmes. The three themes, (i) the use and potential impact of RGCS, (ii) practitioners' beliefs and expectations about the process of delivering RGCS, and (iii) the resources available for practitioners for RGCS, were highly interconnected. Grouping the barriers and enablers by community, practitioner and organisation revealed a preponderance of practitioner barriers and organisational enablers. Enablers were not identified for all of the barriers found in the literature and will need consideration using implementation science and behaviour change theory to develop potential approaches. To ensure the successful delivery of RGCS programmes the need for an implementation plan and relevant implementation strategies has been noted.^25^ Such studies require time, collaboration and funding to have impact.^47^ Further research is required to identify and then test evidence‐informed implementation strategies.

## CONFLICT OF INTEREST

The authors have no conflict of interest to declare.

## Supporting information

Supplementary Material
