# Supplementary material for: Health practitioners' perceptions of the barriers and enablers to the implementation of reproductive genetic carrier screening: A systematic review
Source: Prenat Diagn. 2021 Mar 5;41(6):708–19. doi: 10.1002/pd.5914 (PMC8252081; doi:10.1002/pd.5914)
Supplement: Supplementary file 1 — Supplementary Material [file PD-41-708-s001.pdf]

Supplementary table 1: Detail of barriers and enablers to implementation of RGCS programmes

Theme	Sub theme	Barriers	Enablers
**The use of reproductive carrier screening and the impact it may have**	a) Achieving equitable service provision	Cost of testing<br>Lack of consistency in panels public/private sector	Link with other health interventions<br>Communication with policy makers and other stakeholders
	b) Potential impact (including the offer) on the patient	Possibility of raising women's anxiety<br>Feeling coerced<br>Lack of general public interest in RCS<br>Misperceptions of the 'perfect child'	Informed consent – active participation and understanding implications<br>Decision aids/client education<br>Raise knowledge of general population
**Practitioner beliefs and expectations about delivery of RCS**	a) Practitioner attitudes to and beliefs about RCS	Lack of collective sense of urgency<br>Positive attitude to RCS not followed up by offering RCS in practice<br>Practitioners not interested<br>Variability about who should be tested<br>Concern about offering testing as routine would influence clients' decision to take up offer<br>Concern about false positives/ medicalising pregnancy and perceptions of eugenics<br>Unsure about adult onset conditions	Client request for RCS<br>Experience
	b) Practitioner perceptions of their ability to deliver RCS	GPs – perception of lack of ability to discuss positive results and possibility of termination when women is first pregnant<br>Lack of confidence in offering prenatal genetic advice, what diseases to test for, interpreting the results and explaining results<br>Confusing criteria to guide HCPs makes RCS appear too hard	Obstetricians and gynaecologists were comfortable with offering RCS<br>Training<br>Knowing when to refer on
	c) Practitioner knowledge and support required to deliver RCS	Limited training in genetics leads to lack of underlying knowledge<br>Lack of support for practitioners	Call for training<br>Dissemination of research findings<br>Raise awareness of screening guidelines<br>Increase genetic support for HCPs
	d) Practitioner expectations and external views influencing their decision making	Offers made due to liability and professional obligation i.e. not guided by client need/preference<br>Continual renegotiation of the place of RCS<br>Conflicting advice from different professional bodies	Role of the professional bodies to provide consistent guidance<br>Evidence based implementation including relevance and acceptance by society
**Resources available for practitioners to use for reproductive carrier screening**	a) Provision of counselling including genetic counsellors and other professionals	Access to counselling as a limited resource<br>Concern non-specialist staff would find counselling challenging and underestimate the complexity of counselling	Ensure couples are well informed before a consultation<br>Experience and time for non-specialist staff to develop time efficient counselling skills
	b) Variation in potential models of service provision	GPs-RCS does not fit with GP targets<br>Use of repeat visits to ensure testing does not become routine	Develop GP specialists with screening skills<br>Identify other professions to offer screening e.g. midwives<br>Offer testing outside health care settings<br>Preference to offer screening pre pregnancy, at preconception carrier screening clinics
	c) Non clinical resource barriers	Time<br>Cost to the health system<br>Lack of focus on public health<br>Identifying appropriate measures of effectiveness	Upskilling HCPs (to reduce time)<br>Additional resources<br>Leadership<br>Development of an implementation plan with implementation strategies
